# Dual-energy CT in gout patients: Do all colour-coded lesions actually represent monosodium urate crystals?

**DOI:** 10.1186/s13075-020-02283-z

**Published:** 2020-09-11

**Authors:** Sara Nysom Christiansen, Felix Christoph Müller, Mikkel Østergaard, Ole Slot, Jakob M. Møller, Henrik F. Børgesen, Kasper Kjærulf Gosvig, Lene Terslev

**Affiliations:** 1 Copenhagen Center for Arthritis Research, Center for Rheumatology and Spine Diseases, Rigshospitalet, Glostrup, Valdemar Hansens Vej 17, 2600 Glostrup, Denmark; 2 Department of Clinical Medicine, University of Copenhagen, Copenhagen, Denmark; 3 Department of Radiology, Herlev and Gentofte Hospital, Herlev, Denmark; 4 Siemens Healthineers, Ballerup, Denmark

**Keywords:** Dual-energy CT, Gout, MSU crystals, Property analysis, Artefacts, Specificity

## Abstract

**Background:**

Dual-energy CT (DECT) can acknowledge differences in tissue compositions and can colour-code tissues with specific features including monosodium urate (MSU) crystals. However, when evaluating gout patients, DECT frequently colour-codes material not truly representing MSU crystals and this might lead to misinterpretations. The characteristics of and variations in properties of colour-coded DECT lesions in gout patients have not yet been systematically investigated.

The objective of this study was to evaluate the properties and locations of colour-coded DECT lesions in gout patients.

**Methods:**

DECT of the hands, knees and feet were performed in patients with suspected gout using factory default gout settings, and colour-coded DECT lesions were registered. For each lesion, properties [mean density (mean of Hounsfield Units (HU) at 80 kV and Sn150kV), mean DECT ratio and size] and location were determined. Subgroup analysis was performed post hoc evaluating differences in locations of lesions when divided into *definite MSU depositions* and possibly other lesions.

**Results:**

In total, 4033 lesions were registered in 27 patients (23 gout patients, 3918 lesions; 4 non-gout patients, 115 lesions). In gout patients, lesions had a median density of 160.6 HU and median size of 6 voxels, and DECT ratios showed an approximated normal distribution (mean 1.06, SD 0.10), but with a right heavy tail consistent with the presence of smaller amounts of high effective atomic number lesions (e.g. calcium-containing lesions).

The most common locations of lesions were 1st metatarsophalangeal (MTP1), knee and midtarsal joints along with quadriceps and patella tendons. Subgroup analyses showed that *definite MSU depositions* (large volume, low DECT ratio, high density) had a similar distribution pattern, whereas *possible calcium-containing material* (high DECT ratio) and *non-gout MSU-imitating lesions* (properties as *definite MSU depositions* in non-gout patients) were primarily found in some larger joints (knee, midtarsal and talocrural) and tendons (Achilles and quadriceps). MTP1 joints and patella tendons showed only *definite MSU depositions*.

**Conclusion:**

Colour-coded DECT lesions in gout patients showed heterogeneity in properties and distribution. MTP1 joints and patella tendons exclusively showed *definite MSU deposition*s. Hence, a sole focus on these regions in the evaluation of gout patients may improve the specificity of DECT scans.

## Introduction

Diagnosis of gout is traditionally based on characteristic clinical symptoms combined with elevated plasma urate levels and preferably joint/tophus aspiration with microscopical verification of monosodium urate (MSU) crystals [1]. However, aspiration is not always possible in routine clinical practice, and imaging techniques have gained an increasing role in the diagnosis of gout patients [1]. The latest ACR/EULAR gout classification criteria developed in 2015 for use in clinical trials have also incorporated both ultrasound and dual-energy CT (DECT) as ways to detect MSU depositions in patients [2].

DECT has diagnostic potential in gout as it can automatically colour-code MSU depositions, based on predefined software settings. The technique is based on simultaneous acquisition of images at two different levels of x-ray photon energy (high- and low-kV series). It exploits the physical principle that attenuation of x-ray photons depends not only on the density but also on the effective atomic number (Zeff) of the scanned material [3–5]. Attenuation of x-ray photons is described by CT values and measured in Hounsfield units (HU).

For MSU crystal identification, the DECT scanner needs to distinguish MSU depositions from its surrounding tissues—most importantly calcium-containing material (bones/calcified soft tissues) and soft tissues (tendons/cartilage/synovial tissue). This distinction is achieved through two discriminations. *First*, a discrimination between MSU depositions and calcium-containing materials can be made since the two materials have different DECT ratios. The DECT ratio describes the difference in attenuation at high- and low-kV series, and this difference is material-dependent since high-Zeff materials (such as calcium-containing tissues) attenuate low energy x-ray photons more than low-Zeff materials (such as MSU depositions) [3, 6]. *Second*, a discrimination between MSU depositions and soft tissues (which have similar Zeff) can be made since MSU depositions are generally more dense than soft tissues resulting in a higher mean CT value [3, 6].

**Fig. 1:**
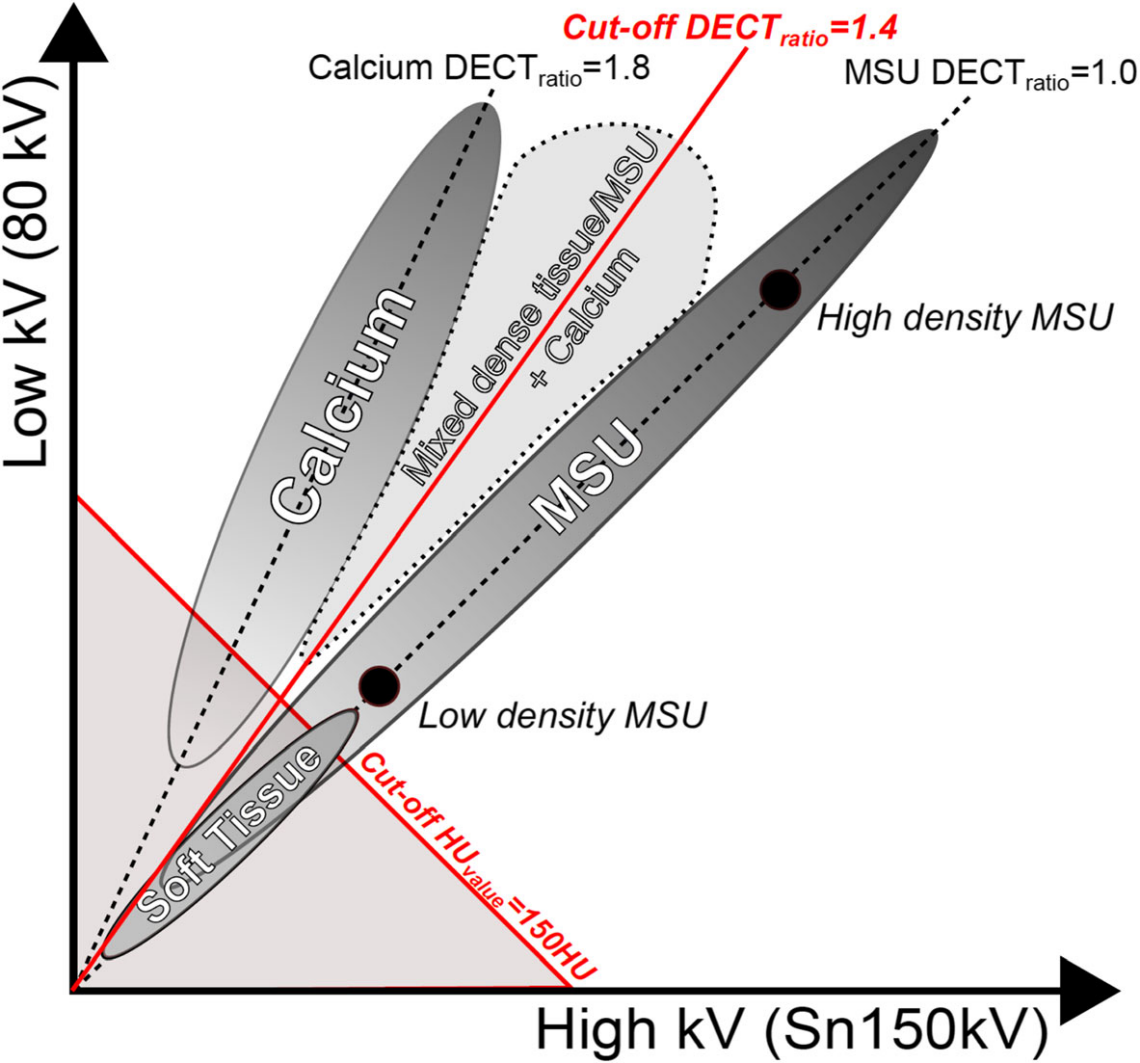
Simplified schematic of algorithm used in DECT scans for the evaluation of gout. The algorithm separates the chemical composition of compounds based on their different attenuations at 80 kV and 150 kV (with additional tin (Sn) filter). Three materials (MSU, soft tissue and calcium) can be differentiated from each other. For simplicity, only the range of different concentrations of pure calcium in water is depicted. Materials above the cut-off DECT_ratio_ are classified as calcium (i.e. material with high Zeff), and materials below the cut-off DECT_ratio_ are classified as MSU depositions and colour-coded green (i.e. materials with low Zeff). A HU cut-off differentiates soft tissues (with Zeff similar to MSU) from MSU depositions. Mixed tissues (such as calcified dense tissues of calcified MSU depositions) may lead to misinterpretations of DECT examinations: as illustrated by the light grey area. DECT, dual-energy computed tomography; MSU, monosodium urate; Zeff, effective atomic number

In DECT scans, the distinction between MSU depositions and surrounding tissues is based on cut-off values read into the DECT software. A DECT ratio cut-off classifies a material as either calcium-containing (attenuation above the cut-off) or MSU depositions (attenuation below the cut-off), and a density cut-off classifies materials as either soft tissues (density below the cut-off) or MSU depositions (density above the cut-off) (Fig. 1). Increasingly compact materials (calcium, MSU and soft tissue) will be located increasingly upward and right on their respective dotted lines in Fig. 1 (as exemplified by high- and low-density MSU). If tissues are increasingly calcified (e.g. calcifications in soft tissues or in MSU depositions), an increased attenuation at the low-kV series will increase their slopes (as seen in the light grey area, Fig. 1). Therefore, definite cut-offs do not apply in patients, since the deposition of both MSU crystals and calcium occurs gradually causing tissues to often contain a mixture of different materials thereby obtaining properties, which can lead to misinterpretations of DECT scans. Furthermore, small lesion artefacts, which occur due to image noise, may obtain properties mimicking MSU depositions thereby also appearing colour-coded on DECT scans leading to misinterpretations [7].

Despite these known tissue features, the characteristics of and variations in properties of colour-coded DECT lesions in gout patients have not yet been systematically investigated.

The primary aim of this study was to evaluate the locations and properties (defined as the DECT ratios, densities and sizes) of colour-coded DECT lesions in gout patients. The secondary aims were, (1) based on lesion properties, to evaluate the likelihood of each colour-coded DECT lesion containing either solely MSU crystals or possibly other materials and (2) to evaluate the distribution of colour-coded DECT lesions with different compositions across joint and tendon regions.

## Patients and methods

### Design and patients

This study was a cross-sectional, observational study. Patients were recruited at the Center for Rheumatology and Spine Diseases, Rigshospitalet, Denmark. DECT examinations were conducted at the Department of Radiology, Herlev Hospital, Denmark. Patients were included prospectively and consecutively. Eligible for inclusion were adult patients (≥ 18 years) referred from primary care or other hospital departments with a clinical suspicion of gout. No exclusion criteria were applied.

### Clinical, laboratory and microscopy assessments

For characterization of the cohort, disease duration and joint/tendon regions ever involved were recorded. Patients reported pain intensity (visual analogue scale) and numbers of joint attacks within the past 12 weeks. Clinical joint examination and laboratory tests were performed. Puncture of a joint/tophus was performed in all patients in a currently/previously inflamed joint or tophus, either as an aspiration of fluid or as a dry needle aspiration [8]. The sample was examined by independent assessors (OS/VF, both certified examiners [9]). If no MSU crystals were identified, the puncture was repeated after 2 weeks. If the joint/tophus aspiration was negative for MSU crystals after the retest, the patients were examined for clinical and laboratory signs of gout as outlined in the ACR/EULAR 2015 gout classification criteria [2].

### Dual-energy CT

DECT examinations were performed using a third-generation dual-source DECT scanner (Siemens Somatom Force; Siemens Healthineers, Forchheim, Germany). Bilateral DECT scans were performed of the knees and feet (scanned bilaterally simultaneous) and hands (separate acquisitions for each side). All scans were obtained without intravenous contrast media using a dedicated DECT protocol: collimation 128 × 0.6 mm (hands), 64 × 0.6 mm (knees/feet); pitch 0.3 (hands), 0.5 (knees/feet); rotation time 0.5 s; CTDI_vol_ 3 mGy (hands), 9.5 mGy (knees), 7.0 mGy (feet), without automatic tube current modulation. Tube voltages were set at 80 kV and 150 kV, the latter with an additional tin filter. Scan time was < 1 min per joint. Transversal series were reconstructed using a quantitative kernelQr40 at a slice thickness of 0.75 mm at 0.5-mm increments and appropriate field of views. Voxel sizes were 0.5 × 0.5 × 0.5 mm (hands), 0.35 × 0.35 × 0.5 mm (knees) and 0.6 × 0.6 × 0.5 mm (feet).

#### DECT image postprocessing

DECT datasets were postprocessed in commercially available postprocessing software Syngo.ViaVB30 (Siemens Healthineers, Forchheim, Germany) using the “Gout” application class at factory default settings including a DECT ratio of 1.4, minimum HU at 150 HU and maximum at 500 HU. Transversal gout series (slice thickness 0.75 mm with 0.5-mm increments) where then exported into a custom MeVisLab-application (MevisLab vers.2.8.2, GmbH, Bremen, Germany).

All colour-coded DECT voxels in the scanned regions were automatically registered. Colour-coded DECT lesions were defined as a 3-dimensional cluster of colour-coded DECT voxels adjacent to each other.

#### Lesion properties

The size, density and DECT ratio of each lesion were automatically recorded. Size was defined as the number of voxels in a lesion. Density was defined as the mean of HU values at high-kV and low-kV for all voxels in the lesion. DECT ratios were defined as HU value at low-kV divided by HU value at high-kV series.

#### Lesion location

The locations of all individual colour-coded DECT lesion in the scanned joint regions in all patients were coded according to a location map (Additional file 1) by a single reader (SNC). Obvious artefacts such as nail-bed or skin artefacts [7] were not included. For reporting data, locations were merged into a reduced set of joint/tendon locations (Fig. 4, Additional file 1).

#### Subgroup analysis

The most important lesions, which may cause misinterpretations when analysing DECT scans, are dense soft tissues in non-relevant locations (e.g. nail-bed artefacts, excluded in our study), calcium-containing tissues (with only a small amount of calcium, e.g. calcified menisci/cartilage), dense tendons (e.g. the Achilles tendon) and small lesion artefacts. Subgroup analyses were performed in order to distinguish colour-coded DECT lesions than *unquestionably* represented MSU depositions from lesions than *could potentially* represent other lesions with the aim of evaluating potential differences in distribution of lesions between these two groups. In order to make these distinctions, we separated lesions into 4 different subgroups, where grouping was performed based on lesion properties. The first group (*possible calcium-containing material*) were defined as the lesions with the highest DECT ratios, which were more likely to contain high-Zeff materials. The second group (*possible dense tendon*) were defined as lesions with low DECT ratios *and* with the lowest densities, which were more likely to represent dense tendons than the remaining lesions. The third group (*possible image noise artefacts*) were defined as the lesions with the lowest sizes, since small lesions are more likely to represent artefacts compared to larger lesions. The fourth group (*definite MSU depositions*) were defined as lesions fulfilling all characteristics of MSU depositions: low DECT ratio, high density and large volume. This combination of material property characteristics applies only to MSU depositions due to the composition of the material, and this lesion type could therefore only represent pure MSU crystals. For non-gout patients (here defined as MSU-negative patients), properties of *non-gout MSU-imitating lesions* (properties as *definite MSU depositions* in non-gout patients) were analysed.

The above-mentioned grouping of lesions was made based on applied cut-off values, and these values were chosen based on the lesion properties found in our dataset. Therefore, the cut-off values were not founded on cut-off values described by others. Three cut-off values were applied to differentiate the four groups: (1) a DECT ratio cut-off—high DECT ratio lesions > mean DECT ratio in large lesions in gout patients + 2 standard deviations (SD); (2) a density cut-off—low-density lesions < lower quartile range (QR) of average HU value in large lesions in gout patients; and (3) a size cut-off—small size lesions < median size of *all lesions*.

### Statistical analysis

Descriptive statistics were used to summarize baseline data and to describe properties of colour-coded DECT lesions. Patients’ baseline characteristics and properties of colour-coded DECT lesions were presented as means with (SD) for normally distributed variables and as medians with ranges and interquartile ranges (IQR) for non-normally distributed variables. Statistical analyses were performed using SAS Enterprise-Guide v.7.15.

## Results

In total, 27 patients were included in the study from December 2017 till September 2019. Of these, 23 were classified as “gout patients” (MSU-positive patients) and 4 patients were classified as “non-gout patients” (negative microscopy for MSU crystals). Table 1 shows the baseline characteristics of the patients. Patients were predominantly males (96%), mean age of > 60 years and with rather long mean disease duration (> 7 years). In total, 4033 colour-coded DECT lesions were registered, where 3918 lesions were found in gout patients (median lesions pr. patient 47) and 115 lesions in non-gout patients.

**Table 1:** Demographic and baseline characteristics (*n* = 27)

		Gout patients (MSU-positive) (***n*** = 23)	Non-gout patients (MSU-negative) (***n*** = 4)
Age, years, mean (SD), [range]		62.6 (12.8) [39–85]	63.5 (7.3) [55–71]
Male sex, no. (%)		22 (96%)	3 (75%)
Calcium pyrophosphate-positive (joint puncture) patients, no. (%)		2 (9%)	3 (75%)
Fulfilment of the ACR/EULAR 2015 gout classification criteria at the time of inclusion or 1 year after inclusion*, no. (%)		23/23 (100%)	0/4 (0%)
Self-reported disease duration, months, median (IQR), [range]		108 (36; 180) [3–456]	84 (39; 138) [6–180]
No. of joint attacks within 12 weeks, median (IQR), [range]		1 (1; 4) [0–12]	2 (1; 4) [0–4]
Self-reported region of pain/joint attacks (ever) (pct.):			
Fore- and midfoot		22 (96%)	4 (100%)
Ankle region (incl. Achilles tendon)		8 (35%)	1 (25%)
Knee		16 (70%)	1 (25%)
Finger and/or wrist		11 (48%)	4 (100%)
Visual analogue scale, pain, 0–100, median (IQR), [range]		35 (10; 60) [5–90]	38 (25; 60) [20–75]
No. of tender joints (0–60), median (IQR), [range]		5 (2; 10), [0–26]	7 (3; 11), [2–11]
No. of swollen joints (0–60), median (IQR), [range]		1 (0; 5), [0–10]	2 (1; 3), [0–3]
P-urate	(mmol/L), mean (SD), [range]	0.50 (0.11) [0.32–0.70]	0.40 (0.08) [0.32–0.50]
	(mg/dL), mean (SD), [range]	8.4 (1.8) [5.4–11.8]	6.7 (1.3) [5.4–8.4]
Number of patients with colour-coded DECT lesions (%)		21/23 (91%)	1/4 (25%)
Colour-coded DECT lesions (*n*)		3918	115
Colour-coded DECT lesions pr. patient, median (IQR), [range]		47 (10; 226), [3–1308]	–

*All gout patients were MSU-positive and therefore also fulfilled the ACR/EULAR classification criteria for gout as MSU crystal identification is a sufficient criterion [2]. MSU-negative patients were evaluated according to the remaining clinical and laboratory criteria but excluding the imaging criteria [2]. DECT, dual-energy CT; ACR/EULAR, American College of Rheumatology/European League Against Rheumatism; SD, standard deviation; IQR, interquartile range; MSU, monosodium urate crystals; P-urate, plasma urate

### Analysis of colour-coded DECT lesions in gout patients

#### Properties of colour-coded DECT lesions in gout patients

**Fig. 2:**
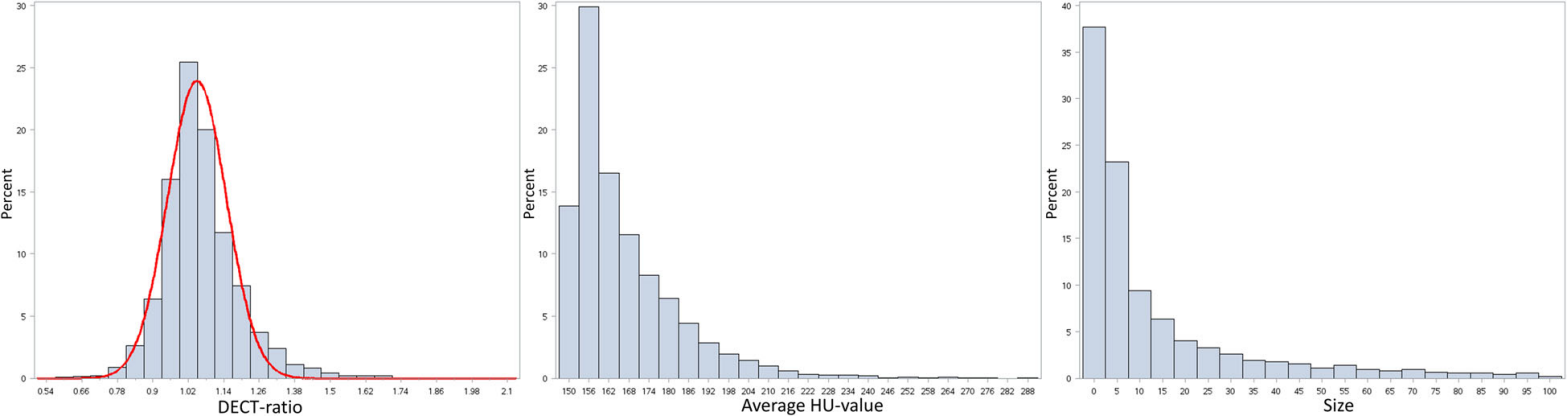
Distributions of lesion properties. Properties of colour-coded DECT lesions in gout patients. The distributions of DECT ratios have been overlaid with a normal distribution curve with a mean at the local maxima at a DECT ratio of 1.06 and a standard deviation estimated from points below this mean to be 0.10. Notice that the right tail on the DECT ratios is heavy with more lesions having a high DECT ratio than expected by a Gaussian distribution in agreement with a mixture of monosodium urate depositions and calcium-containing material. DECT, dual-energy computed tomography; DECT ratio, HU at 80 kV/HU at 150 kV (with tin filter); HU, Hounsfield units; size, numbers of voxels

**Fig. 3:**
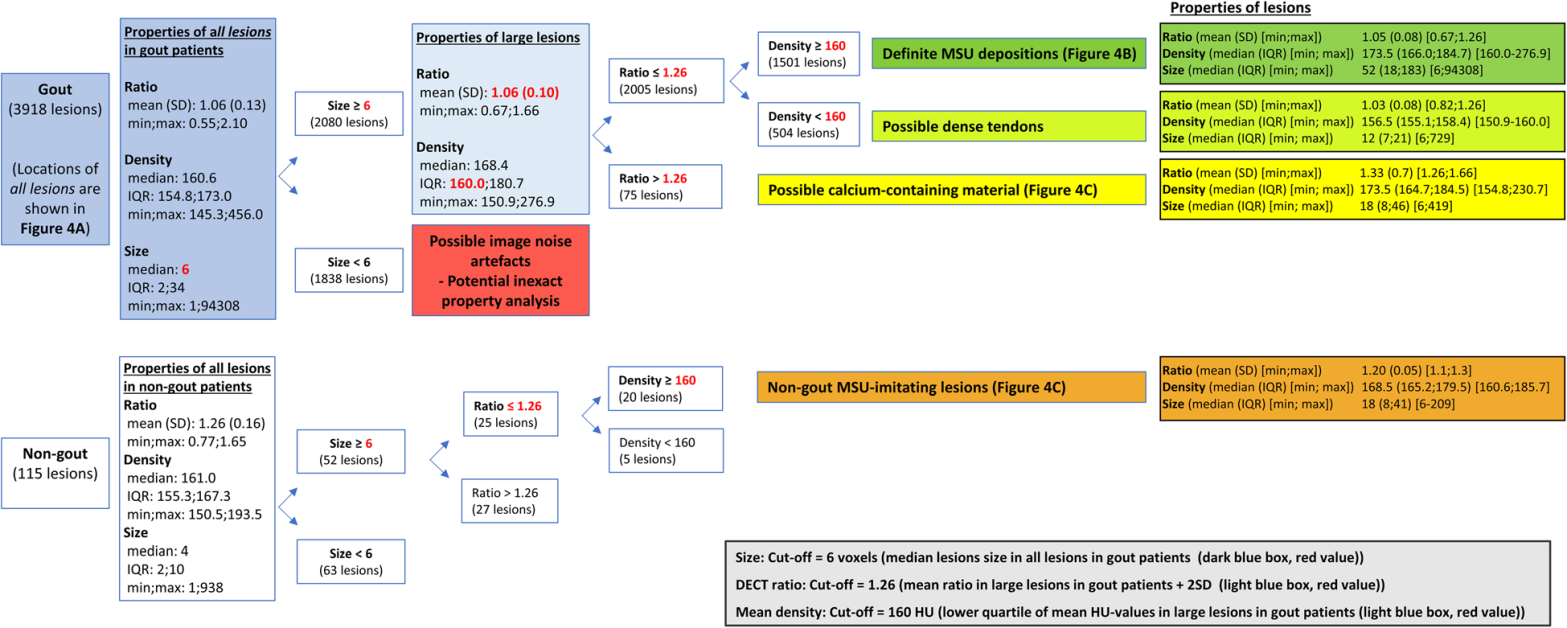
Properties of colour-coded DECT lesions in gout and non-gout patients. DECT, dual-energy computed tomography; HU, Hounsfield units; ratios, DECT ratios (HU at 80 kV/HU at 150 kV with tin filter); density, (HU at 80 kV + HU at 150 kV with tin filter)/2; size, numbers of voxels; SD, standard deviation; IQR, interquartile range; MSU, monosodium urate

The DECT ratios of colour-coded DECT lesions in gout patients approximated a normal distribution with a mean of 1.06 (SD 0.13), but the distribution showed a heavy right tail (Figs. 2 and 3 (blue box)), consistent with MSU depositions being the primary component but also containing a small amount of high-Zeff lesions (calcium-containing lesions). The average density of lesions showed a right-skewed distribution with a minimum of 150 HU (due to factory default gout settings), a median value of 160.6 HU and very few lesions with an average density of > 200 HU (Figs. 2 and 3). Sizes of analysed lesions also showed a right-skewed distribution with a median size of 6 voxels (Figs. 2 and 3).

#### Locations of colour-coded DECT lesions in gout patients

**Fig. 4:**
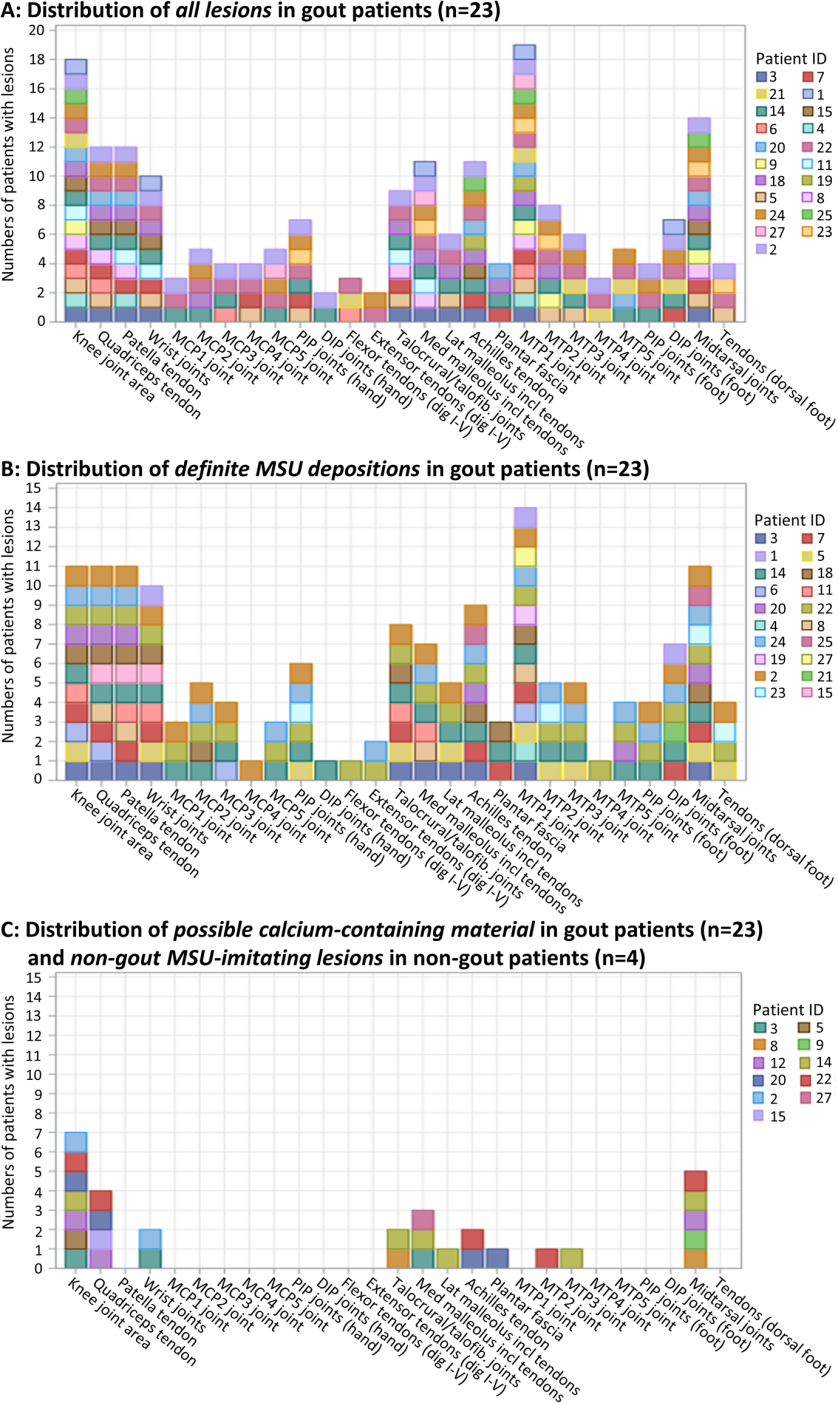
MCP, metacarpophalangeal joints; PIP, proximal interphalangeal joints; DIP, distal interphalangeal joints; MTP, metatarsophalangeal joints; MSU, monosodium urate

Colour-coded DECT lesions in gout patients (labelled *all lesions*) were seen in all analysed regions, but with heterogeneity in distribution (Fig. 4a). Common locations (defined as locations where lesions were seen in > 50% of patients) for *all* colour-coded DECT lesions included the knee (78% of patients), the MTP1 (83% of patients) and the midtarsal joints (61% of patients) along with the quadriceps and patella tendons (both 52% of patients) (Fig. 4a).

### Subgroup analyses of colour-coded DECT lesions in all patients

*Possible image noise artefacts* (size < 6 voxels) in gout patients included 1838 lesions (Fig. 3, red box). Of the larger lesions, 1501 lesions fulfilled the characteristics of *definite MSU depositions* (≥ 6 voxels, DECT ratio ≤ 1.26, density ≥ 160 HU, Fig. 3, dark green box), 504 lesions fulfilled the characteristics of *possible dense tendons* (DECT ratio ≤ 1.26, density < 160HU, Fig. 3, light green box), and 75 lesions fulfilled the characteristics of *possible calcium-containing material* (DECT ratio > 1.26, Fig. 3, yellow box). In non-gout patients, 20 lesions fulfilled the characteristics of *non-gout MSU-imitating lesions* (properties as *definite MSU depositions*, Fig. 3, orange box).

**Fig. 5:**
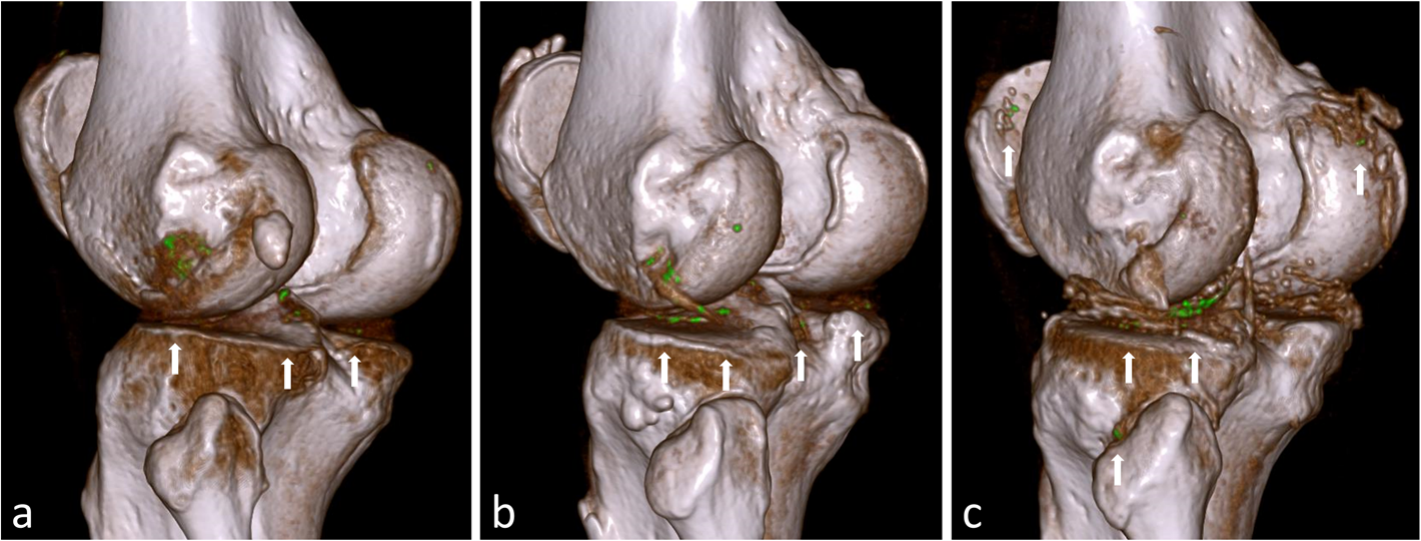
DECT images showing examples of colour-coded DECT lesions in knee joints. **a**–**c** Dual-energy CT (DECT) scans of the left knee joints in three different patients showing colour-coded DECT lesions with similar appearances and locations. Analysis of the DECT ratios revealed that **a** is dominated by *definite MSU depositions*, while **b** and **c** are dominated by likely calcium-containing depositions. **a** *Definite MSU depositions* in a gout patient. A 54-year-old gout patient with no comorbidities. DECT revealed 58 colour-coded DECT lesions characterized by a low mean DECT ratio (1.01, SD 0.09) consistent with the lesions representing pure MSU depositions. **b** Calcium-containing depositions in a gout patient. A 67-year-old gout patient with comorbidities in the form of obesity and knee osteoarthritis. DECT revealed 39 colour-coded DECT lesions characterized by a high mean DECT ratio (1.22, SD 0.16) consistent with lesions representing calcified tissues (possibly calcifications in the menisci/cartilage and/or calcified MSU depositions). **c** Calcium-containing depositions in a non-gout patient. A 56-year-old non-gout patient with comorbidities in the form of obesity and myocardial disease. The plasma urate level was 0.29 mmol/L (4.9 mg/dL), and joint puncture showed an absence of MSU crystals but a presence of multiple calcium pyrophosphate (CPP) crystals. DECT revealed 57 colour-coded DECT lesions characterized by a high mean DECT ratio (1.24, SD 0.17) consistent with lesions representing calcified tissues (possibly calcifications in the menisci/cartilage and/or CPP crystal depositions)

*Definite MSU depositions* were found with similar distribution when compared to *all lesions* in gout patients, with the same five most common locations (Fig. 4b). *Possible dense tendon* lesions had a mean HU value of 156.5 HU—markedly higher than expected for dense tendons—and lesion locations were similar to *definite MSU depositions* (data not shown), indicating that they primarily consisted of pure MSU depositions. In contrast, *possible calcium-containing material* and *non-gout MSU-imitating lesions* had distinctly different properties (DECT ratios 1.33 and 1.20, respectively (Fig. 3, yellow and orange boxes)). Furthermore, the locations of these lesions were different from *definite MSU depositions*, as they were primarily found in some larger weight-bearing joints (knee, midtarsal and talocrural including malleolus regions) and in certain tendons (Achilles and quadriceps), whereas no such lesions were found in MTP1 joints and patella tendons (Fig. 4c). Figure 5 shows examples of colour-coded DECT lesions in the knee joints of three different patients from our study population.

#### Common MSU non-specific locations

Common (involved in > 50% of patients) MSU non-specific locations included the knee and midtarsal joints and the quadriceps tendon since these locations showed both *definite MSU depositions* and *uncertain lesions* (Fig. 4a vs. c). A colour-coded lesion in one of these locations could therefore represent either pure MSU depositions or possibly other lesions.

#### Common MSU-specific locations

Common (involved in > 50% of patients) MSU-specific locations included only the MTP1 joint and the patella tendon since these locations exclusively showed *definite MSU depositions* (Fig. 4b vs. c). Any larger (≥ 6 voxels) colour-coded DECT lesion in one of these two locations represented *definite MSU depositions* (328/328 lesions, 100%). All gout patients with colour-coded DECT lesions (21/21 patients, 100%) had lesions in either the MTP1 joint or the patella tendon (Fig. 4a). Hence, for gout patients in our cohort, colour-coded DECT lesions in the MTP1 joint and patella tendon had excellent sensitivity and specificity for diagnosing patients with *definite MSU depositions*.

## Discussion

This study is the first to evaluate the specific properties of colour-coded DECT lesions in gout patients. Our study demonstrates that colour-coded DECT lesions in gout patients are heterogeneous in properties. Some colour-coded DECT lesions fulfil all characteristics of pure MSU depositions and therefore can be classified as “definite”, whereas other lesions have a higher DECT ratio and therefore must contain high-Zeff materials such as calcium and do not necessarily contain MSU crystals.

Most previous studies have not focused on the properties of colour-coded DECT lesions, but rather on the diagnostic accuracy of DECT scans in predicting gout at a patient level. Several studies have evaluated the specificity of colour-coded DECT lesions using either MSU microscopy [10–14] or other criteria as reference standards (e.g. physician evaluated diagnosis, ACR 1977 gout classification criteria) [14–18]. A recent systematic literature review calculated the pooled sensitivity and specificity of DECT examinations to be 0.81 and 0.91, respectively [19]. However, specificities for colour-coded DECT lesions varied markedly in these studies (0.48–1.00) [10–19] reflecting a varying proportion of false-positive findings, which could indicate that not all colour-coded DECT lesions truly represent MSU depositions. Bongartz et al. [11] showed that DECT demonstrated colour-coded lesions in 7 out of 41 non-gout patients, and all of these lesions were found within the cartilage/menisci in patients with knee osteoarthritis. The authors therefore concluded that DECT may have limited specificity in knee osteoarthritis [11]. In our patient cohort, only one non-gout patient showed colour-coded DECT lesions, and therefore, no conclusions can be drawn regarding diagnostic sensitivity or specificity of DECT scans at the patient level. However, the property analysis showed that the colour-coded DECT lesions in this non-gout patient had markedly different properties when compared to lesions in gout patients, where especially the mean DECT ratio was higher (1.26 vs. 1.06). This non-gout patient had a joint puncture negative for MSU crystals but positive for calcium pyrophosphate (CPP) crystals, and the patient did not fulfil the ACR/EULAR 2015 gout classification criteria neither at time of inclusion nor after 1 year. It is therefore possible that the colour-coded DECT lesions in this patient in fact represented CPP crystals rather than MSU crystals, but future studies are needed to investigate the discriminatory ability of DECT to distinguish these two crystal types.

Another explanation for colour-coded DECT lesions in non-gout patients might be small MSU depositions causing only “subclinical disease”, and some authors suggest this as an explanation for the false-positive findings [7]. However, this statement is not based on the assessment of lesion properties. When applying a DECT ratio cut-off well above 1.0, which is used in most studies [11, 12, 14, 18], one would expect some of the colour-coded DECT lesions to be other materials than MSU depositions (e.g. calcium-containing), since pure MSU crystals show equal HU values when scanned at high- and low-kV series (DECT ratio ≈ 1) [6]. The approximated but heavy right tailed normal distribution of DECT ratios in our study underlines that some colour-coded DECT lesions do contain high-Zeff materials and not necessarily represent MSU depositions.

In our study, we did not assess the effect of altering neither the DECT ratio cut-off nor the HU threshold, as the aim of our study was to evaluate colour-coded DECT lesions using factory default gout settings. However, two recent studies have investigated the effect of changing postprocessing protocols when performing DECT examinations [20, 21]. Both studies investigated the effect of lowering the threshold of attenuation from 150 HU to 120 HU [20] or 130 HU [21], respectively. Both studies found this to result in an improved visualization of MSU depositions, but lowering of HU threshold also resulted in an increased amount of artefacts, especially artefacts in well-known locations such as tendons, nail-beds and skin [20]. One of the studies found that the artefacts lead to misclassifications of patients [21], whereas the other study found specificity of DECT examination to remain unchanged [20].

Colour-coded DECT lesions were in our study most commonly found in the knee, MTP1 and midtarsal joints along with the quadriceps and patella tendons. These lesion locations are partly in line with the findings from another study evaluating the distribution of colour-coded DECT lesions [22]. This study also found the MTP1 joint to be the most common site for lesions involved in 57% of patients, while the knee and tarsal joints were among the 10 most prevalent locations [22]. Tendon lesions were seen more than twice as often in the Achilles tendon (36%) than in the quadriceps (16%) or patella tendons (12%) [22], whereas our study showed the regions to be involved equally frequent (52%, 52% and 48%, respectively).

Our subgroup analyses revealed that some common locations for colour-coded lesions (knee joint, midtarsal joints and quadriceps tendon) showed both *definite MSU depositions* and *uncertain lesions*, whereas two common locations for colour-coded lesions (MTP1 joint and patella tendon) exclusively showed *definite MSU depositions*. In line with our results, Bongartz et al. [11] found the anatomic region used for diagnostic assessment of importance for the overall specificity of the DECT examination. Bongartz et al. focused their primary analysis on an index joint, where DECT scans had a sensitivity of 0.90 and a specificity of 0.83 when compared to MSU crystal microscopy. Secondary analyses expanded the examination to include all green pixels of the scanned area (primarily feet and knees) resulting in a higher sensitivity (0.95) but a significant drop in specificity (0.56) [11].

In order to avoid false-positive DECT examinations, a specificity-optimized evaluation set for reading DECT scans of gout patients would be beneficial. One approach could be to simply lower the DECT ratio in the factory default gout settings, thereby excluding more high-Zeff lesions. However, our study does not support this strategy, since this would not have excluded the *non-gout MSU-imitating lesions*. Instead, a sole focus on the MTP1 joint or the patella tendon would have increased the specificity without reducing sensitivity of DECT examination in gout patients. In order to avoid image noise artefacts, which are often seen when analysing small lesions [7], we furthermore—based on lesion properties—introduced a size criterion where we excluded lesions < 6 voxels. For measurements in a two-dimensional plane, this would equal ≥ 1 mm in lesion diameter. This proposed size criterion is identical to previously proposed size criteria. Mallinson et al. [7] proposed several potential artefacts, and these artefacts are also included in the ACR/EULAR 2015 gout classification criteria. Here it is stated that “nailbed, submillimeter, skin, motion, beam hardening, and vascular artifacts should not be interpreted as DECT evidence of urate deposition” [2], where submillimeter artefacts are described as “colouring of single pixels or areas smaller than 1 mm” [7]. The size criterion proposed in our study did not change our findings at the patient level, since all patients—which had colour-coded DECT lesions—also had lesions > 5 voxels/≥ 1 mm. A specificity-optimized evaluation set for DECT examinations based on our patient cohort would therefore include only the evaluation of large lesions (diameter ≥ 1 mm) located in either the MTP1 joints or the patella tendons, but this evaluation set requires further prospective validation.

The strengths of our study include that quantitative property analysis for all colour-coded DECT lesions was combined with location analysis in a large number of joint/tendon regions enabling us to determine the distribution pattern of lesions differing in DECT properties. Since quantitative lesion properties were automatically included in the analyses (except for obvious artefacts like nail-bed artefacts), no reader variance was introduced, thereby making interreader reliability assessment unnecessary. Diagnosis of all patients was based on MSU crystal microscopy thereby securing that all study-defined gout patients truly had gout. The DECT scanner used in our study (Siemens Somatom Force) was a third-generation dual-source DECT scanner, which has higher spectral separation and higher precision [23, 24] compared with DECT scanners used in other studies [10–18]. Image noise can partly be reduced through improved postprocessing software and through increased spectral separation in the newer generations of DECT scanners which by itself reduces image noise in dual-energy calculations [23]. Emerging techniques may allow for dramatically improved spectral imaging, as it is seen in photon-counting CT, where every pixel gives exact physical material and/or tissue information [25]. In gout, this results in images with a finely detailed punctate pattern of MSU crystal depositions in contrast to the clump-like appearance on DECT [26].

A major limitation of this study was the small number of patients included. Although the numbers of lesions were high (4033 lesions), they were derived from a small number of patients (*n* = 22), where especially the numbers of non-gout patients showing colour-coded DECT lesions were low (*n* = 1). However, the aim of this study was not to establish the diagnostic accuracy of DECT at the patient level but to evaluate the properties of colour-coded DECT lesions in gout patients. A thorough investigation of the properties of colour-coded DECT lesions across joint/tendon regions in non-gout patients compared to such lesions in gout patients should be evaluated in future studies.

## Conclusions

Our study demonstrates that colour-coded DECT lesions in gout patients both represent pure MSU depositions and depositions likely to be calcium-containing, and the characteristics that may help to differentiate these two types of DECT lesions are outlined. In the current gout patient cohort, colour-coded DECT lesions at the MTP1 joint and patella tendon were exclusively pure MSU depositions. All gout patients had lesions in one or both of these locations. A sole focus on these regions when diagnosing gout patients may therefore improve specificity without reducing sensitivity of DECT scans. However, further studies are needed in order to establish if DECT assessment of these two regions reliably distinguishes gout from non-gout patients.

## Supplementary information

**Additional file 1.** Location map for registration of colour-coded DECT lesions.

## Data Availability

The dataset used and/or analysed during the current study are available from the corresponding author on reasonable request.

## Abbreviations

ACR: American College of Rheumatology
CPP: Calcium pyrophosphate
DECT: Dual-energy computed tomography
DIP: Distal interphalangeal
EULAR: European League Against Rheumatism
HU: Hounsfield units
IQR: Interquartile ranges
PIP: Proximal interphalangeal
MCP: Metacarpophalangeal
MTP: Metatarsophalangeal
SD: Standard deviation
Zeff: Effective atomic number
