# Supplementary material for: Dual-energy CT in gout patients: Do all colour-coded lesions actually represent monosodium urate crystals?
Source: Arthritis Res Ther. 2020 Sep 11;22:212. doi: 10.1186/s13075-020-02283-z (PMC7488422; doi:10.1186/s13075-020-02283-z)
Supplement: Supplementary file 1 — Additional file 1. Location map for registration of colour-coded DECT lesions. [file 13075_2020_2283_MOESM1_ESM.docx]

**Additional file 1: Location map for registration of colour-coded DECT lesions.**


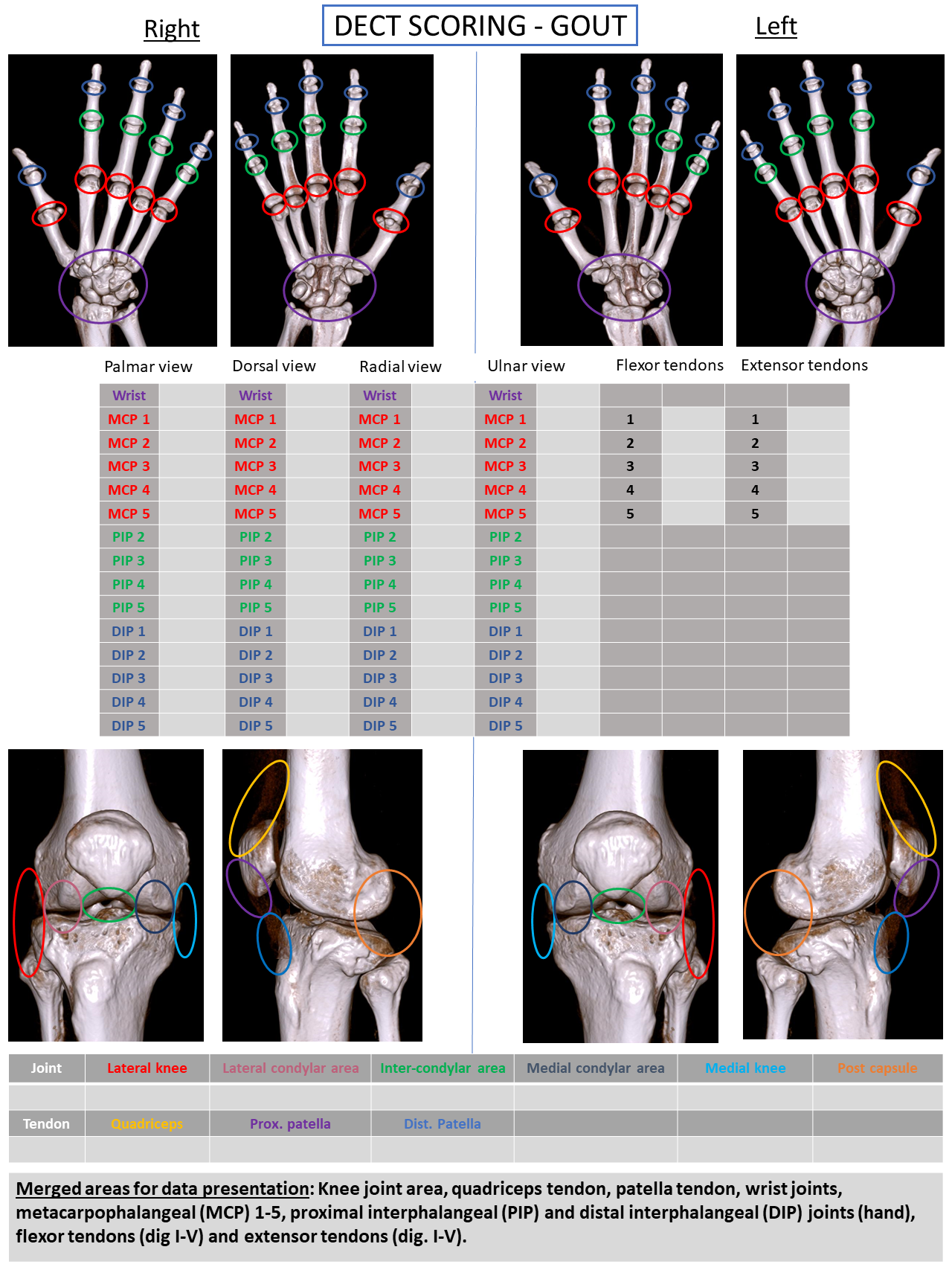


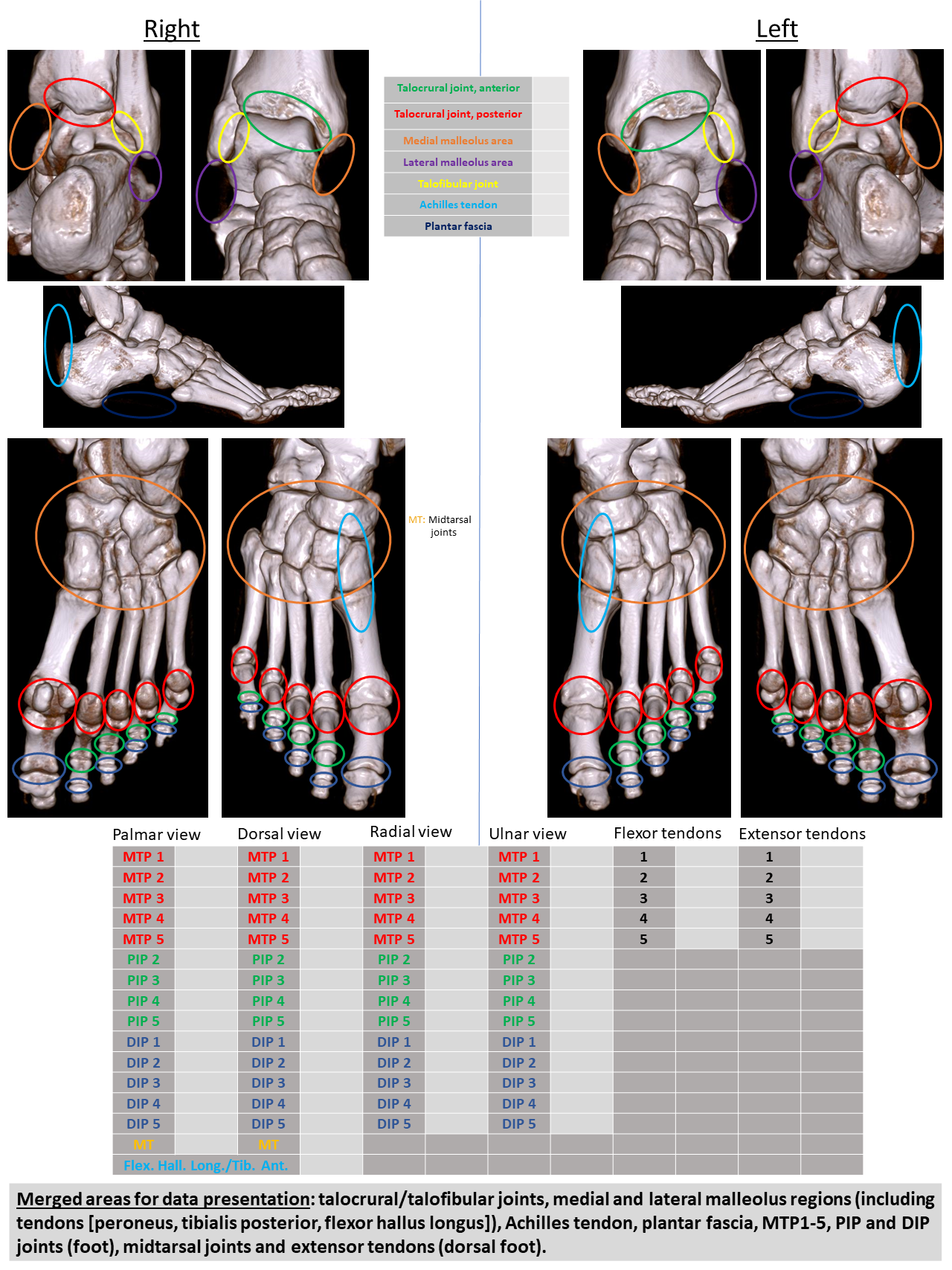


DECT, dual-energy computed tomography; MCP, metacarpophalangeal; MTP, metatarsophalangeal; PIP, proximal interphalangeal joint; DIP, distal interphalangeal joint; MT; midtarsal joints; Flex. Hall. Long; flexor hallus longus tendon; Tib. Ant, tibialis anterior tendon.
